# Selective sorption of uranium from aqueous solution by graphene oxide-modified materials

**DOI:** 10.1007/s10967-018-5741-4

**Published:** 2018-02-17

**Authors:** H. Mohamud, P. Ivanov, B. C. Russell, P. H. Regan, N. I. Ward

**Affiliations:** 1 Department of Chemistry, University of Surrey, Guildford, GU2 7XH UK; 2 National Physical Laboratory, Hampton Road, Teddington, TW11 OLW UK; 3 Department of Physics, University of Surrey, Guildford, GU2 7XH UK

**Keywords:** Sorption, Uranium, Carboxyl-functionalised graphene oxide, Selectivity, ICP-MS

## Abstract

The effect of competing ions on the sorption behaviour of uranium onto carboxyl-functionalised graphene oxide (COOH-GO) were studied in batch experiments in comparison to graphene oxide (GO) and graphite. The effect of increasing the abundance of select chemical functional groups, such as carboxyl groups, on the selectivity of U sorption was investigated. In the course of the study, COOH-GO demonstrated superior performance as a sorbent material for the selective removal of uranyl ions from aqueous solution with a distribution coefficient of 3.72 ± 0.19 × 10^3^ mL g^−1^ in comparison to 3.97 ± 0.5 × 10^2^ and 2.68 ± 0.2 × 10^2^ mL g^−1^ for GO and graphite, respectively.

**Electronic supplementary material:**

The online version of this article (10.1007/s10967-018-5741-4) contains supplementary material, which is available to authorized users.

## Introduction

Uranium belongs to the actinide series and has three naturally occurring radioisotopes: ^238^U (*T*_1/2_ = 4.468 × 10^9^ ± 0.005 y), ^234^U (*T*_1/2_ = 2.455 × 10^5^ ± 0.006 y) and ^235^U (*T*_1/2_ = 7.1 × 10^8^ ± 0.011 y) with an average abundance of 2.4 mg kg^−1^ in the Earth’s crust [1, 2]. The high prevalence of uranium and its radiotoxicity makes it a vital radionuclide to monitor in the environment [3]. Furthermore, numerous studies have highlighted the importance of developing rapid and effective treatment processes for aqueous nuclear waste produced in activities related to the nuclear fuel cycle [4–6]. Existing treatment processes, which are currently used at an industrial scale to remove uranium from aqueous nuclear waste commonly involve ion-exchange, co-precipitation and solvent extraction [7–10]. However, these processes typically exhibit low selectivity when their distribution co-efficients (*K*_d_) are reported. Moreover, they often display slow sorption kinetics for target long-lived radionuclides, especially in the presence of competing ions [11]. Thus, alternative techniques capable of selective and rapid removal of uranium from aqueous solution would be of significant value.

One such technique is sorption which has been widely used due to its ease of operation, simplicity and limited use of solvents [12]. Recent studies have demonstrated that nanomaterials, specifically graphene oxide (GO), outperform traditional sorbent materials, such as bentonite and activated carbon, by exhibiting higher loading capacities and efficiencies for uranium removal [13]. This is believed to be due to the exceptional intrinsic properties of GO, including an extremely high contact surface, plus a wide range of chemical functionalities [14, 15]. As a result, the presence of selective surface functional groups on GO, such as, carboxyls and hydroxyls, enable for the sorption of uranyl species through surface complexation [16]. This has been demonstrated by Li et al. who have reported the use of GO for uranium removal and determined the maximum sorption capacity to be 299 mg g^−1^ at pH 4 [17]. The efficiency of GO for uranium removal has been found to be improved by the addition of larger chelating ligands on the surface of GO. For instance, Wang et al. have shown functionalising GO with amidoxime led to an increased sorption capacity of 398.4 mg g^−1^ at pH 6 [18]. In addition, selectivity for uranium removal was found to be enhanced in comparison to GO when the material was exposed to simulated seawater comprising of Mg, Ca, Ba and Sr [18]. Therefore, these results demonstrate the effectiveness of both GO and surface-modified GO for uranium removal. Furthermore, such studies also illustrate the need for additional investigation into the effect of increasing the abundance of complexing groups, such as carboxyls, on the selective removal of uranium.

The aim of this study is to synthesise carboxyl-functionalised graphene oxide materials (COOH-GO) designed with a high affinity towards the sorption of long-lived actinides, focusing on uranium. The sorption behavior of COOH-GO was investigated and compared to GO and graphite, in the form of batch sorption studies, which included studying the effect of pH, contact time and competing ions prior to inductively coupled plasma mass spectrometry (ICP-MS) analysis. Moreover, each of the sorbent materials were further analysed using a series of surface characterisation techniques, such as Fourier transform infrared spectroscopy and Raman spectroscopy. In addition, thermogravimetric analysis and a methylene blue colourimetric assay were performed to attain a full characterisation profile of each material to assess their suitability for use in radionuclide sorption, waste processing and immobilisation.

## Experimental

### Reagents and materials

All chemical reagents used were of analytical grade and purchased from Sigma Aldrich (Poole, UK). As-received natural graphite flakes (< 45 μm, grade 230, Asbury Graphite Mill Ltd) were used as the starting material to prepare graphene oxide (GO). For ICP-MS measurements, a 100 mg mL^−1^ stock standard solution of uranium in 2% HNO_3_ (Fisher Scientific, Loughborough, UK) and multi-element standard (MES) solution (Fisher Scientific, Loughborough, UK) containing 5000–20,000 μg mL^−1^ of Mg, Co, Zn, Sr, Pb, Th and U in 2% HNO_3_ were used (see Table S1). Solutions were diluted with ultrapure deionised (DI) water obtained using an ELGA purelab flex water purification system (ELGA, Veolia Water, Marlow, UK, 18 MΩcm, < 5 ppb Total Organic Carbon). The pH was measured with a digital pH/ISE meter (Orion Star A214, Thermo Scientific, UK)

### Preparation of GO

GO was synthesised from natural graphite according to the modified Hummers method [19–21]. Briefly, 1 g of graphite was added to 120 mL of sulphuric acid (H_2_SO_4_, 98%) and 0.5 g of sodium nitrate (NaNO_3_, 99%), which was continuously stirred on a magnetic hot plate at 300 rpm for 1 h and cooled to 20 °C using a water bath. Next, 6 g of potassium permanganate (KMnO_4_, 99%) was slowly added and the resulting mixture was left to stir overnight at 35 °C. A solution of 10 mL of hydrogen peroxide (H_2_O_2_, 35%) in 400 mL of ice was next added resulting in a bright yellow precipitate.

For work-up, the remaining precipitate was collected, diluted with 500 mL of 0.5 M hydrochloric acid (HCl, 99%) solution and purified by repeated washing with DI water and centrifugation (4000 rpm, 20 min) until the pH of the supernatant was neutral. To achieve nano-sized flakes of GO, a series of sonication treatments with an ultrasonic bath (Ultrawave U300H) were completed for 1 h and the resulting product was freeze-dried to obtain graphene oxide.

### Preparation of COOH–GO

COOH-GO was synthesised by reducing the hydroxyl groups present in GO to carboxyl groups [22, 23]. In a typical procedure, 0.05 g of GO in 50 mL of DI water was sonicated for 30 min. Next, 1.2 g of chloroacetic acid (ClCH_2_COOH, 99%) and 1 g of sodium hydroxide (NaOH, 99%) were added to the GO solution and sonicated for 3 h. The resulting black COOH-GO mixture was neutralised with 0.5 M HCl solution and purified by repeated washing with DI water and centrifugation (2000 rpm, 10 min). Finally, the resulting product was freeze-dried to obtain carboxylated graphene oxide.

### Characterisation techniques

**Fig. 1:**
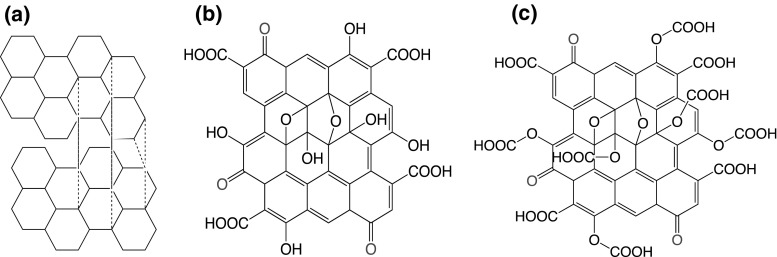
The proposed chemical structures of **a** graphite, **b** GO and **c** COOH-GO [20, 22]

Figure 1 shows the proposed structure of the materials under investigation. Structural analysis was completed by characterising the samples by Fourier transform infrared spectroscopy (FTIR) and Raman spectroscopy. FTIR spectra of solid powdered samples were recorded on a Cary 670 FTIR spectrometer using attenuated total reflectance (Agilent Technologies, UK). Raman spectra of all samples were obtained on a DXR high resolution Raman microscope (Thermo Scientific, UK) equipped with an Ar laser (irradiation wavelength 532 nm, 10 mW laser power, 0.7 μm spot size, ×50 microscope objective, 10 s collection exposure, 32 scans) and an average of three sample spots were selected for study with the data collected analysed using OMNIC™ software. Elemental analysis was conducted on a CE440 elemental analyser (Exeter Analytical, USA). Thermal analysis was obtained by thermogravimetric analysis (TGA) and was carried out on a TGA Q500 (TA Instruments, USA). Samples were placed into platinium crucibles (1–2 mg) and heated from ambient temperature to 900 °C at a heating rate of 10 °C min^−1^ under a N_2_ gas flow.

### Methylene blue assay

The quantification of the total abundance of carboxyl groups functionalised to graphite, GO and COOH-GO was determined by the published method of Imani et al. and involved the use of a colorimetric-based assay with the dye molecule methylene blue (MB) [24, 25]. Initially, a standard calibration curve of aqueous MB solutions (0.2–5 μg mL^−1^) was prepared and recorded at 664 nm in 0.1 M sodium bicarbonate (NaHCO_3_, 99%) buffer at pH 8. Next for a typical MB assay, 2 μg mL^−1^ of aqueous MB solution was added to 5 mg of graphite, graphene oxide and carboxylated graphene oxide, respectively and incubated for 15 min. After centrifugation for 5 min at 4500 rpm, 1 mL of the supernatant was collected and then analysed via UV–vis spectrophotometry (Biochrom Libra S80, UK) with the absorbance recorded at 664 nm.

### Batch sorption experiments

**Fig. 2:**
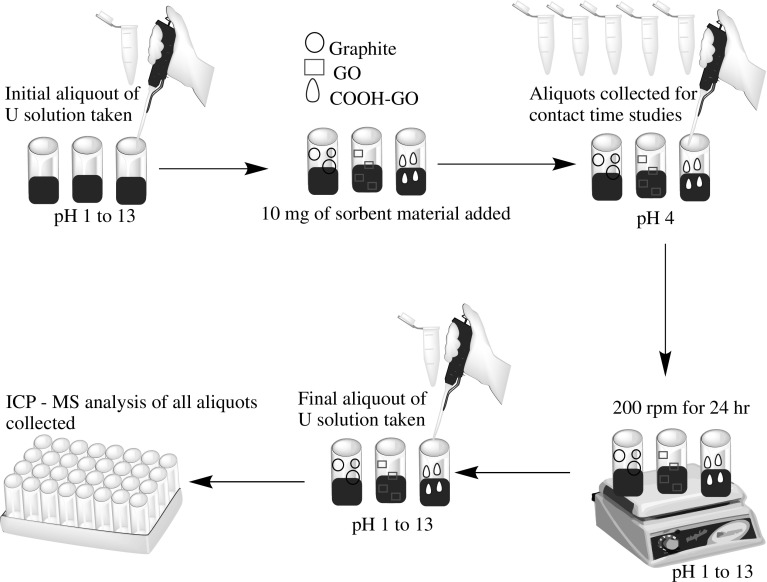
Schematic representation of the single-component batch studies completed for the sorption of U onto graphite, GO and COOH-GO

The sorption of U onto graphite, GO and COOH-GO materials was investigated in batch experiments as ilustrated in Fig. 2. To test the effect of pH, a series of 10 mL U solutions (10 μg mL^−1^) were prepared in 15 mL centrifuge tubes, which were pH adjusted from pH 1–13 with 0.01–1.00 M solutions of HNO_3_ and NaOH, respectively. For contact time studies, the pH was adjusted to pH 4 with 0.01 M HNO_3_ and timed aliquots were collected from 5 to 140 min. In a typical sorption experiment, an initial aliquot was taken of the prepared solutions to determine the initial concentration of U. This was then subsequently followed by the addition of 10 mg of sorbent material to the pH-adjusted suspensions. The samples were shaken, left for 24 h to equilibrate and a final sample aliquot was collected.

To test the effect of competing ions, the sorption experiment was repeated with 10 mL of diluted multi-element standard (MES) solutions (see Table S1) at pH 4. The U and MES concentrations in both the initial and final aliquots collected for the single and multi-component batch studies were analysed using an inductively coupled plasma mass spectrometer (ICP-MS) (Agilent 8800, Agilent Technologies, UK) [26]. The instrument was fitted with a quartz double-pass spray chamber and a MicroMist nebuliser (Glass Expansion, Melbourne, Australia) and nickel sample and skimmer cones (Crawford Scientific, South Lanarkshire, UK). The instrument was tuned daily using a mixed 1 μg mL^−1^ standard tuning solution.

The percentage of U and other elements of interest sorbed onto graphite, GO and COOH-GO in the batch studies was determined by Eq. (1) with the corresponding distribution co-efficient, *K*_d_, determined by Eq. (2).

1 $${\text{Sorption}} \left( \% \right) = 100 - \left( {\frac{\text{CPSf}}{\text{CPSi}}} \right) \times 100$$

2 $$K_{\text{d}} ({\text{mLg}}^{ - 1} ) = \left( {\frac{{{\text{CPSi}} - {\text{CPSf}}}}{\text{CPSf}}} \right) \times \left( {\frac{V}{m}} \right)$$

where CPSi refers to the initial counts per second detected prior to the addition of the sorbent sample by ICP-MS, and CPSf refers to the final counts per second detected. *V* refers to the volume of standard solution used (U or MES) in mL and *m* refers to the mass of sorbent material used in mg.

## Results and discussion

### Structural analysis

**Fig. 3:**
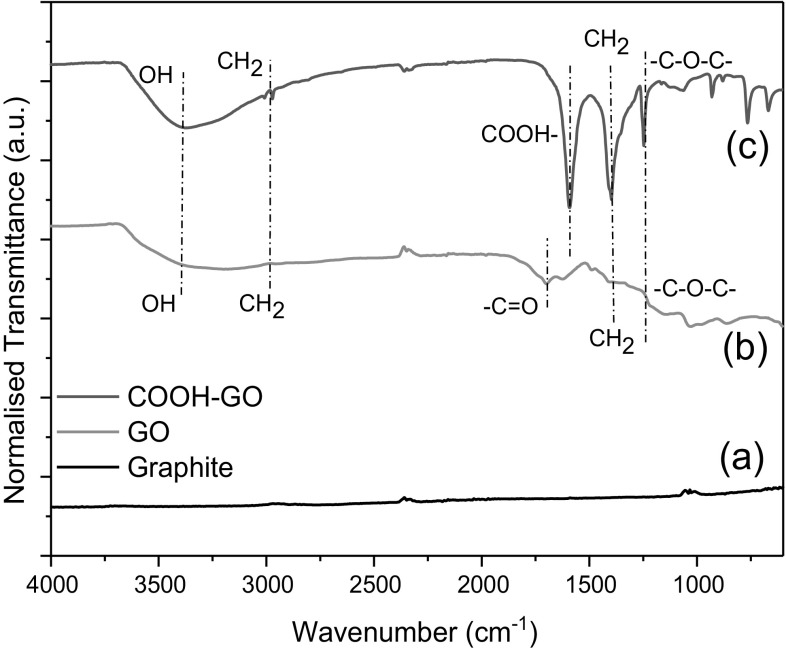
Surface functionalities identified onto **a** graphite, **b** GO and **c** COOH-GO samples by FTIR spectroscopy

FTIR spectroscopy was used to identify key surface functional groups present in graphite, GO and COOH-GO. As depicted in Fig. 3a, graphite had no significant characteristic absorption peaks identified.

GO exhibited characteristic absorption peaks at 3407.3 cm^−1^ due to O–H stretching. Moreover, C=O stretches and C–O–C stretches were found at the following adsorption bands, 1733.2 and 1027.8 cm^−1^, respectively [27, 28]. Further treatment of GO with chloroacetic acid led to the introduction of a new COOH adsorption band at 1644 cm^−1^ [24]. The discovery of this new adsorption band in conjuction with an enhanced O–H absorption peak at 3320.3 cm^−1^ demonstrates the successful introduction of a greater abundance of COOH groups to the surface of graphene oxide to produce COOH-GO.

**Fig. 4:**
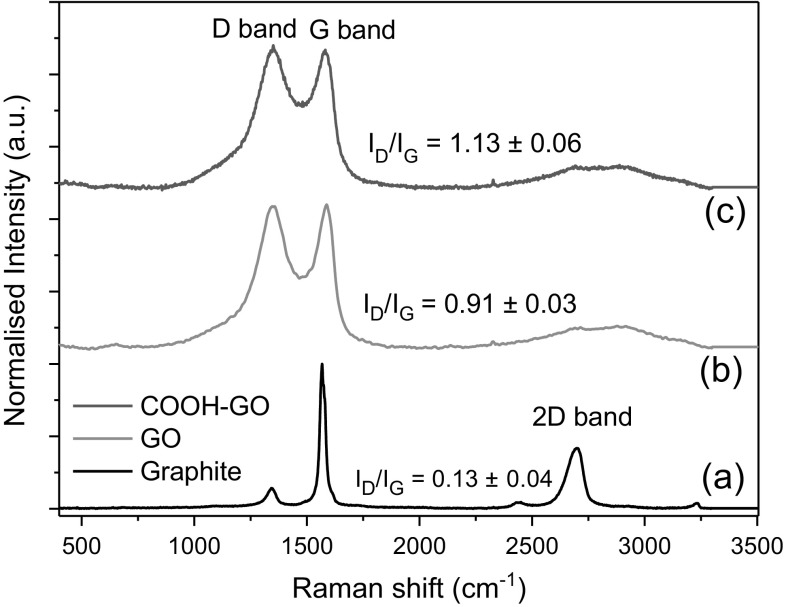
Raman spectra of **a** graphite, **b** GO and **c** COOH-GO with their corresponding I_D_/I_G_ ratios. Values reported as mean ± SD where *n* = 3

Raman spectroscopy is a common technique used to analyse carbon-based materials e.g., carbon nanotubes, graphene and fullerenes [29, 30]. In this study, the technique was used to compare the varying degree of functionalisation attributed to graphite, to that of as-prepared GO and COOH-GO (Fig. 4b, c) [31]. In addition, the intensity ratio between the D and G band (I_D_/I_G_) was also evaluated to monitor the number of sp^2^ i.e., aromatic domains present in the samples [32, 33]. The Raman spectra of GO and COOH-GO showed the presence of two characterisitc bands associated with aromatic hydrocarbon materials: a strong signal for the D band at 1350.6 and 1348.8 cm^−1^, plus an intense signal for the G band at 1585.6 and 1578.5 cm^−1^, respectively.

The increase in signal observed in the D band for GO and COOH-GO indicates the successful transformation of the sp^3^ domains, typically present in graphite, into sp^2^ domains [33]. The corresponding I_D_/I_G_ ratios also confirms this finding with the value increasing from 0.13 ± 0.04 for graphite to 0.91 ± 0.03 and 1.13 ± 0.06 for GO and COOH-GO, respectively. Thus, demonstrating the effective introduction of new surface chemical functional groups to GO and COOH-GO.

### Thermal analysis

**Fig. 5:**
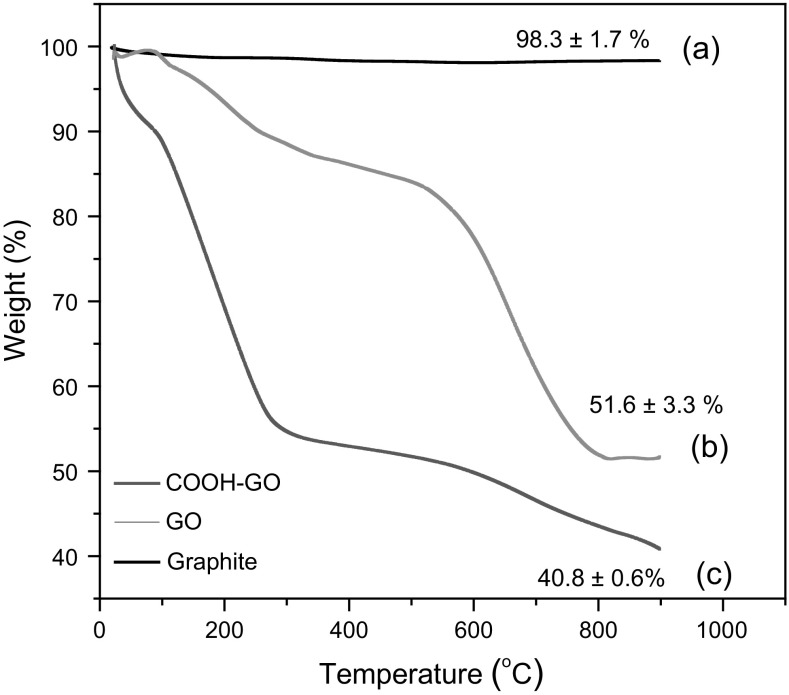
TGA plots of **a** graphite, **b** GO and **c** COOH-GO

Thermogravimetric analysis (TGA) was used to determine the thermal stability of graphite, GO and COOH-GO. Figure 5a illustrates the exceptional thermal stability of graphite with minimal weight loss observed after thermal treatment to 900 °C [34, 35]. The TGA plot of GO indicates that its thermal decomposition occurs primarily through a three-step process [20, 36]. Initially, minimal weight loss is observed from ambient temperature to 130 °C due to the loss of water. This is then followed by a second loss from 180 to 280 °C, which is attributed to the loss of oxygen containing functional groups, such as, hydroxyl and carboxyl groups.

Finally, the third decomposition step observed was from 400 to 790 °C, which was due to the loss of the carbonyl groups present in the sp^2^ carbon network. Figure 5c shows the TGA plot of COOH-GO and illustrates that its thermal decomposition occurs through a similar mechanism as that for GO. However, a sharper loss was observed from 180 to 280 °C, which implies that a greater number of oxygen-containing functional groups, such as, carboxyl groups are present in COOH-GO. This finding agrees well with the reported literature and illustrates the success of the chloroacetic acid/NaOH treatment in introducing carboxyl groups to the surface of the COOH-GO material [37].

### Quantitative analysis of carboxyl groups

**Fig. 6:**
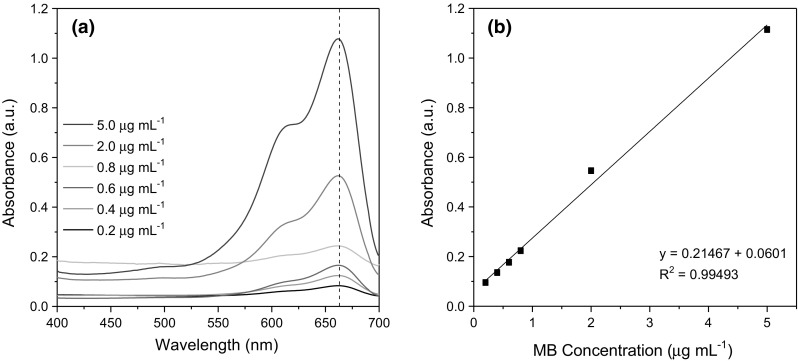
**a** Combined absorbance spectra of MB solutions with increasing concentrations (0.2–5.0 μg mL^−1^). **b** Calibration curve of MB at *λ*_max_ = 664 nm

A methylene blue (MB) colourimetric assay was performed to quantitatively determine the carboxyl content of graphite, GO and COOH-GO (Fig. 6). The assay works on the principle that methylene blue reduces to leucomethylene blue in the presence of samples containing COOH groups [24]. This results in a colour change, which can be monitored by UV–vis spectrophotometry. As a result, the total amount of COOH groups (μmol mg^−1^) present in each sample was determined by measuring the absorbance value of the sample after the addition of MB and comparing that to the absorbance value recorded for the reagent blank (2 μg mL^−1^).

Table 1 highlights the results for the elemental analysis and the MB assay of graphite, GO and COOH-GO. The results showed that, for each sample preparation procedure, the COOH-content increased from 0.0006 ± 0.0002 μmol mg^−1^ for graphite to 0.103 ± 0.003 μmol mg^−1^ for COOH-GO. This data supports the experimental results obtained for the other complementary characterisation techniques i.e., FTIR and TGA analysis.

**Table 1:** Elemental analysis of graphite, GO and COOH-GO and the quantification of carboxyl groups (COOH) based on the MB assay

Sample	Elemental analysis^a^		M_COOH_ (μmol mg^−1^)^b^
	C(%)	O(%)	
Graphite	97.5 ± 0.4	2.4 ± 1.5	0.0006 ± 0.0002
GO	55.8 ± 0.6	42.6 ± 2.4	0.059 ± 0.008
COOH-GO	75.3 ± 0.3	23.6 ± 0.8	0.103 ± 0.003

^a^Values reported as mean ± SD where *n* = 2

^b^Values reported as mean ± SD where *n* = 5

### Uranium sorption studies

#### Effect of pH

**Fig. 7:**
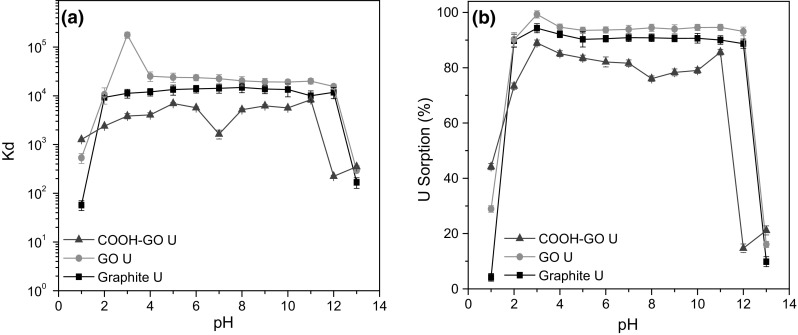
**a** Effect of pH on *K*_d_ values and **b** U sorption onto graphite, GO and COOH-GO (Experimental conditions: U concentration = 10 μg mL^−1^, mass of adsorbent = 10 mg, volume = 10 mL, pH 1–13)

Solution acidity can strongly affect radionuclide speciation and therefore has a significant impact on the sorption process and efficiency of the sorbent materials. Thus, for this study, the effect of pH on the retention of U by graphite, GO and COOH-GO, was evaluated over a pH range from 1 to 13 (Fig. 7a, b). The general trend observed for the three sorbent materials, was for increased U sorption between pH 2 and 11, which is consistent with data previously reported [38, 39].

Of particular note is the trend observed at pH 1, with the *K*_d_ values (mL g^−1^) and sorption (%) attained for U increasing in the following order for the sorbent materials investigated: COOH-GO > GO > graphite. This suggests that the presence of carboxyl groups had an impact on U sorption at significantly low pH levels. This is most likely due to the negatively charged surface of COOH-GO and GO [40] initiating electrostatic interactions with the positively charged U(VI) species, UO_2_^2+^, typically found in solution at low pH which has been reported by Xie et al. [38].

In contrast, at pH 10 and higher, it was observed that the performance of all three sorbent materials decreased considerably. This was believed to be due to the formation of negatively charged and stable uranyl carbonate complexes e.g., [UO_2_(CO_3_)_3_]^4−^ in solution, which has been previously observed to adversely impact U sorption for similar sorbent systems [41, 42]. Thus, it is likely that electrostatic repulsion between the negative U(VI) species and the negatively charged material surface was observed at high pH conditions.

Overall, the optimal condition for U sorption was at pH 3, with GO being the best performing sorbent material, reporting a *K*_d_ value of 1.8 ± 0.11 × 10^5^ mL g^−1^ and 98.7 ± 1.3% U sorption. Furthermore, COOH-GO and graphite displayed *K*_d_ values of 3.8 ± 0.17 × 10^3^ and 1.1 ± 0.04 × 10^4^ mL g^−1^, respectively with the U sorption for COOH-GO and graphite being 88.9 ± 1.9 and 94.3 ± 1.7%, respectively. These results indicate that each of the sorbent materials are suitable for the removal of uranium in solution and are consistent with the performance typically observed for commerical ion-exchange resins [43].

#### Effect of contact time

**Fig. 8:**
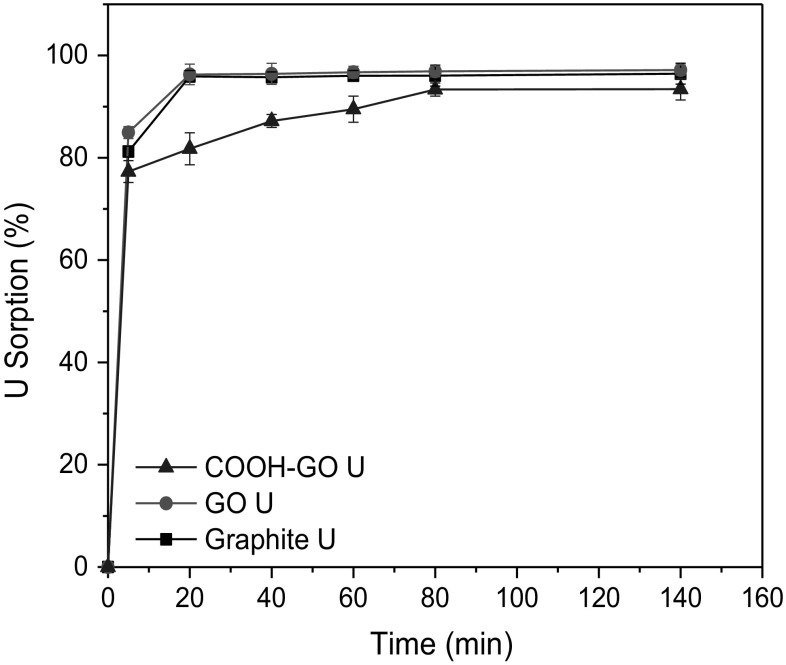
Effect of contact time on U sorption onto graphite, GO and COOH-GO. (Experimental conditions: U concentration = 10 μg mL^−1^, mass of adsorbent = 10 mg, volume = 10 mL, pH 4)

A series of time-controlled studies were performed on graphite, GO and COOH-GO from contact times of 5–140 min at pH 4. Figure 8 illustrates the rapid kinetics of the sorption process for all of the materials studied, with 77.3 ± 1.2–84.9 ± 1.1% U sorption observed within 5 min. These results compare well with those reported in the literature [17] and illustrates that the time required to reach equilibrium is 80 min with over 93.4 ± 2.1% U sorption attainable for COOH-GO.

#### Effect of competing ions

**Fig. 9:**
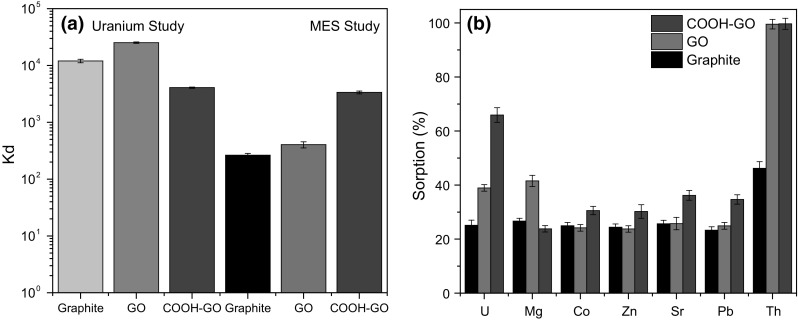
**a** Effect on uranium selectivity by the presence of competing ions using graphite, GO and COOH-GO. **b** Sorption percentage of U over Mg, Co, Zn, Sr, Pb and Th onto graphite, GO and COOH-GO. (Experimental conditions: U concentration = 50 μg mL^−1^, mass of adsorbent = 10 mg, volume = 10 mL, pH 4)

The selective removal of U was investigated by exposing graphite, GO and COOH-GO to a multi-element standard (MES) solution comprising of Mg, Co, Zn, Sr, Pb and Th. It was found that the performance of the graphite and GO sorbent materials were adversely impacted by the presence of competing ions (MES study) with the *K*_d_ values decreasing considerably from 1.17 ± 0.084 × 10^4^ to 2.68 ± 0.2 × 10^2^ mL g^−1^ for graphite (Fig. 9a). For GO, the *K*_d_ values decreased from 2.4 ± 0.07 × 10^4^ to 3.97 ± 0.5 × 10^2^ mL g^−1^, respectively.

In contrast, it was seen that COOH-GO was the only sorbent material to consistently retain U. The reported *K*_d_ values for COOH-GO remained steady, decreasing from 4.11 ± 0.1 × 10^3^ to 3.72 ± 0.19 × 10^3^ mL g^−1^ after competing ions had been introduced into aqueous solution. This indicates that the presence of carboxyl groups on the surface of the COOH-GO material may influence selectivity towards U [44].

The effect on U sorption (%) by the presence of competing ions was also determined. Figure 9b reveals that COOH-GO has a higher selectivity towards the actinides present in the multi-element tracer solutions with over 65.9 ± 2.7% of U retained in comparison to 38.9 ± 1.2% for GO and 25.1 ± 1.9% for graphite. While, Th was consisitently retained by both GO and COOH-GO at over 99.8%, which is in agreement with previously published studies [45, 46]. As a result, it can be seen that the consistently high *K*_d_ and actinide sorption (%) values shown by COOH-GO makes it a promising sorbent material for selectively removing U from contaminated aqueous nuclear waste.

### Sorption isotherms

**Fig. 10:**
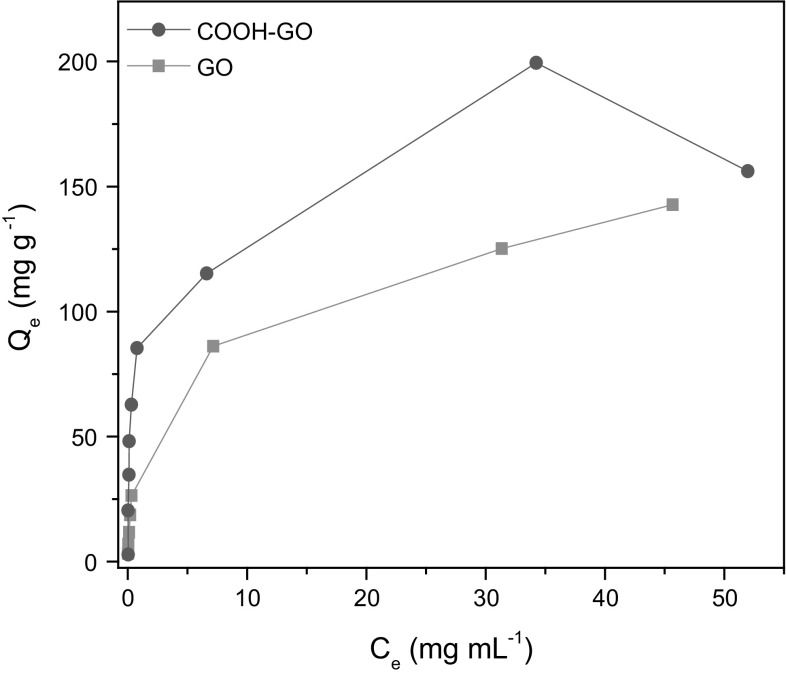
U sorption isotherm for GO and COOH-GO. (Experimental conditions: U concentration = 0.1–60 mg mL^−1^, mass of adsorbent = 10 mg, volume = 10 mL, pH 4)

Sorption isotherms of GO and COOH-GO (Fig. 10) were also investigated by varying the U concentration from 0.1 to 60 mg mL^−1^ and determining the subsequent U capacity. These results were further analysed and characterised by plotting Langmuir and Freundlich isotherm models as depicted in Figs. S1 and S2. It was found from these plots that the Langmuir isotherm model fits the sorption data best. This implies that U sorption by GO and COOH-GO occurs mainly by the formation of a monolayer of U on the sorbent material [13].

Table 2 shows that the maximum sorption capacity (*Q*_max_) of GO and COOH-GO at pH 4 were 142.25 and 169.20 mg g^−1^, respectively. The capacities obtained demonstrate that COOH-GO is more effective in extracting U from solution than GO and the results attained are comparable to those typically observed in GO-based sorbent materials (Table 3). Moreover, the *Q*_max_ value for GO and COOH-GO are considerably higher than those observed for other common sorbent materials such as carbon nanotubes and activated carbon.

**Table 2:** The parameters for the Langmuir and Freundlich isotherm models of U sorption onto GO and COOH-GO

Sample	Langmuir			Freundlich		
	*Q*_max_ (mg g^−1^)	*K*_L_ (mL mg^−1^)	*R* ^2^	*K*_F_ (mL mg^−1^)	*n*	*R* ^2^
GO	142.25	0.527	0.9913	4.856	1.823	0.9125
COOH-GO	169.20	1.310	0.9809	6.384	3.800	0.9299

**Table 3:** Comparison of the U sorption capacities of GO and COOH-GO with other sorbent materials

Sorbents	Experimental conditions	*Q*_max_ (mg g^−1^)	Reference
COOH-GO	pH = 4, *T* = 293 K, equilibrium time (min) = 80	169.2	This study
GO	pH = 4, *T* = 293 K, equilibrium time (min) = 80	142.3	This study
GO	pH = 5, *T* = 293 K, equilibrium time (min) = 60	122.4	[47]
Reduced GO	pH = 4, *T* = 293 K, equilibrium time (min) = n/a	74.1	[39]
Cyclodextrin-modified GO	pH = 5, *T* = 288 K, equilibrium time (min) = n/a	97.3	[48]
Multi-walled carbon nanotubes (MWCNTs)	pH = 5, *T* = 318 K, equilibrium time (min) = 60	39.5	[49]
Activated carbon	pH = 3, *T* = 293 K, equilibrium time (min) = 180	28.3	[50]
Silica-coated Fe_3_O_4_ nanoparticles	pH = 6, *T* = 293 K, equilibrium time (min) = 180	52.4	[51]

## Conclusions

This study demonstrates the capabilities of carboxyl-functionalised graphene oxide (COOH-GO) sorbent materials for selectively removing uranium from aqueous solution. The distribution coefficient is considerably higher than that observed for graphene oxide (GO) and graphite, with a value of 3.72 ± 0.19 × 10^3^ mL g^−1^ under optimal pH conditions. Morever, COOH-GO has a higher sorption capacity for U (*Q*_max_ = 169.20 mg g^−1^) and shows a greater selectivity towards U with 65.9 ± 2.7% retained in the presence of competing ions in comparison to the 38.9 ± 1.2% observed for GO. These enhanced values are likely due to the effect of the presence of selective surface groups, such as, carboxyls. Surface functionalisation analysis of the sorbent materials was performed by FTIR, Raman, TGA and MB colourimetric techniques. The results collected confirmed that sample preparation via the modified Hummers method and chloroacetic acid/NaOH treatment lead to a greater abundance of COOH surface groups being present on GO and COOH-GO.

Future work will involve the completion of reusability studies in real sample matrices and desorption studies to test the efficiency of COOH-GO. It is believed these additional studies will prove to be beneficial in further demonstrating the suitability of this sorbent material for selective actinide removal from aqueous solutions.

## Electronic supplementary material

Below is the link to the electronic supplementary material.

Supplementary material 1 (DOCX 237 kb)
