# Supplementary material for: Selective sorption of uranium from aqueous solution by graphene oxide-modified materials
Source: J Radioanal Nucl Chem. 2018 Feb 17;316(2):839–48. doi: 10.1007/s10967-018-5741-4 (PMC5920007; doi:10.1007/s10967-018-5741-4)
Supplement: Supplementary file 1 — Supplementary material 1 (DOCX 237 kb) [file 10967_2018_5741_MOESM1_ESM.docx]

**Supplementary Information for**

**Selective sorption of uranium from aqueous solution by graphene oxide-modified materials**

**H. Mohamud^1, 2^, P. Ivanov^2^, B. C. Russell^2^, P. H. Regan^2, 3^, N. I. Ward^1^**

*^1^Department of Chemistry, University of Surrey, Senate House, Stag Hill Campus, Guildford, GU2 7XH, United Kingdom*

*^2^Nuclear Metrology Group, National Physical Laboratory, Hampton Road, Teddington, TW11 OLW, United Kingdom*

*^3^Department of Physics, University of Surrey, Senate House, Stag Hill Campus, Guildford, GU2 7XH, United Kingdom*

**SI 1. Individual element concentrations used to prepare the multi-element standard (MES) solution**

| **Element** | **Undiluted concentration (μg mL^-1^)** | **Diluted concentration (μg mL^-1^)** |
| --- | --- | --- |
| Mg | 10000 | 100 |
| Co | 5000 | 50 |
| Zn | 20000 | 200 |
| Sr | 5000 | 50 |
| Pb | 10000 | 100 |
| Th | 5000 | 50 |
| U | 5000 | 50 |

**Table S1** Composition of multi-element standard (MES) solution before and after dilution.

**SI 2. Langmuir and Freundlich isotherm model**

The maximum sorption capacity of single-solute systems can be described by equilibrium sorption isotherm models. These models are typically expressed by constants relating to the surface and binding properties of the sorbent material being characterised. The most widely used sorption model used for this purpose are the Langmuir and Freundlich models. The Langmuir model is based on the assumption that each active site present in the homogenous sorbent material can hold the solute without a reaction being required to take place [1]. Whereas, the Freundlich model is based on the assumption that multilayer sorption processes can occur on heterogeneous sorbent materials [2].

The linearised form of the Langmuir model and Freundlich model are as follows:

$\frac{Ce}{Qe}= \frac{1}{Qmax.KL}+ \frac{Ce}{Qmax}$ (1)

$In\left( Qe \right)= In\left( KF \right)+ \frac{1}{n} x In(Ce)$ (2)

where *Q_e_* (mg g^-1^) and *C_e_* (mg mL^-1^) refer to the sorption capacity and uranium concentration at equilibrium, respectively. *K_L_* (mL mg^-1^) refers to the Langmuir constant which describes the sorption energy of the system. *Q_max_* (mg g^-1^) refers to the maximum sorption capacity of the sorbent material for the solute. *K_F_* (mL mg^-1^) refers to the Freundlich constant which describes the sorption capacity and *1/n* refers to the sorption intensity.

**Fig. S1** The Langmuir (a) and Freundlich (b) isotherm plot for the removal of U on GO. (Experimental conditions: U concentration = 0.1 - 60 mg mL^-1^, mass of adsorbent = 10 mg, volume = 10 mL, pH 4)

**Fig. S2** The Langmuir (a) and Freundlich (b) isotherm plot for the removal of U on COOH-GO. (Experimental conditions: U concentration = 0.1 - 60 mg mL^-1^, mass of adsorbent = 10 mg, volume = 10 mL, pH 4)

References

[1] Günay, A., Arslankaya, E. and Tosun, I., 2007. Lead removal from aqueous solution by natural and pretreated clinoptilolite: adsorption equilibrium and kinetics. J Haz Mat, 146: 362-371.

[2] LeVan, M.D. and Vermeulen, T., 1981. Binary Langmuir and Freundlich isotherms for ideal adsorbed solutions. J. Phys. Chem., 85:3247-3250.
